# Supplementary material for: Adhesion GPCR Gpr126 (Adgrg6) Expression Profiling in Zebrafish, Mouse, and Human Kidney
Source: Cells. 2023 Aug 2;12(15):1988. doi: 10.3390/cells12151988 (PMC10417176; doi:10.3390/cells12151988)
Supplement: Supplementary file 1 [file cells-12-01988-s001.zip › CazorlaV¿ózquezS_supplementary figures_R1_marked.pdf]

cells

MDPI

Supplementary information

Adhesion GPCR Gpr126 (Adgrg6) Expression Profiling in Zebrafish, Mouse, and Human Kidney

Salvador Cazorla-Vázquez [1], Peter Kösters [1], Simone Bertz [2], Frederick Pfister [3], Christoph Daniel [3], Mark Dedden [4], Sebastian Zundler [4], Tilman Jobst-Schwan [5,6], Kerstin Amann [3], and Felix B. Engel [1,*]

[1] Experimental Renal and Cardiovascular Research, Department of Nephropathology, Institute of Pathology, Friedrich-Alexander-Universität Erlangen-Nürnberg (FAU), 91054 Erlangen, Germany.
[2] Institute of Pathology, University Hospital Erlangen, Friedrich-Alexander- Universität Erlangen-Nürnberg (FAU), 91054 Erlangen, Germany.
[3] Department of Nephropathology, Institute of Pathology, University Hospital Erlangen, Friedrich-Alexander- Universität Erlangen-Nürnberg (FAU), 91054 Erlangen, Germany.
[4] Department of Medicine 1, University Hospital Erlangen, Friedrich-Alexander-Universität Erlangen-Nürnberg (FAU), 91054 Erlangen, Germany.
[5] Department of Nephrology and Hypertension, Friedrich-Alexander-Universität Erlangen-Nürnberg (FAU), 91054 Erlangen, Germany.
[6] Research Center On Rare Kidney Diseases (RECORD), University Hospital Erlangen, Friedrich-Alexander-University Erlangen-Nürnberg (FAU), 91054 Erlangen, Germany.

* Correspondence: felix.engel@uk-erlangen.de

Citation: To be added by editorial staff during production.

Academic Editor: Firstname Lastname

Received: date
Accepted: date
Published: date

Publisher's Note: MDPI stays neutral with regard to jurisdictional claims in published maps and institutional affiliations.

Copyright: © 2022 by the authors. Submitted for possible open access publication under the terms and conditions of the Creative Commons Attribution (CC BY) license (https://creativecommons.org/licenses/by/4.0/).

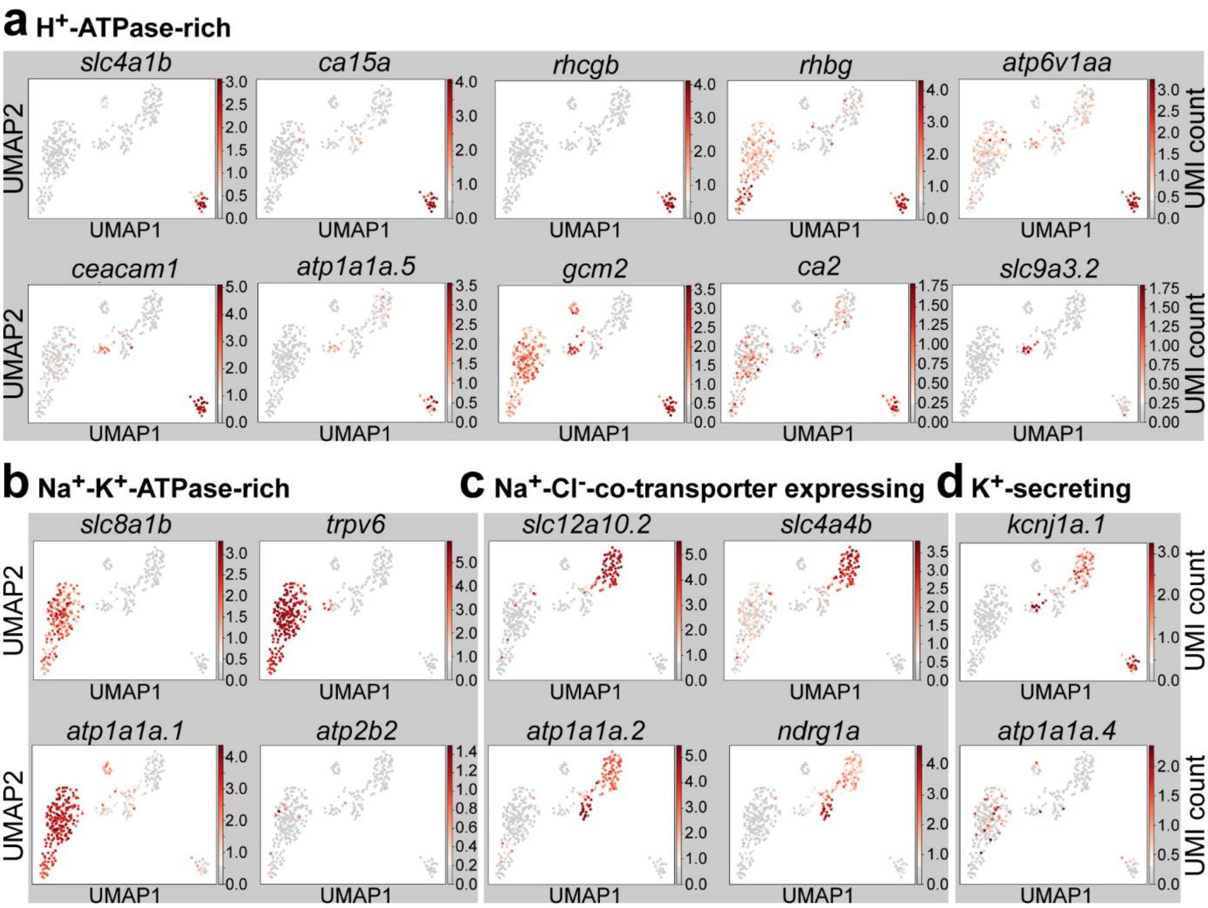

Figure S1. *gpr126* expression is detected in a sub-cluster of ionocytes. (**a-d**) UMAP plot of marker genes for ionocytes, whereby the normalized and scaled UMI (Unique Molecular Identifier) counts are colored in a scale from 0 UMI counts (gray) to the highest UMI count (dark red). The depicted genes are markers for (**a**) H^+-ATPase-rich ionocytes (**b**) Na^+-K^+-ATPase-rich ionocytes (**c**) Na^+-Cl^--co-transporter expressing ionocytes (**d**) K^+-secreting ionocytes.

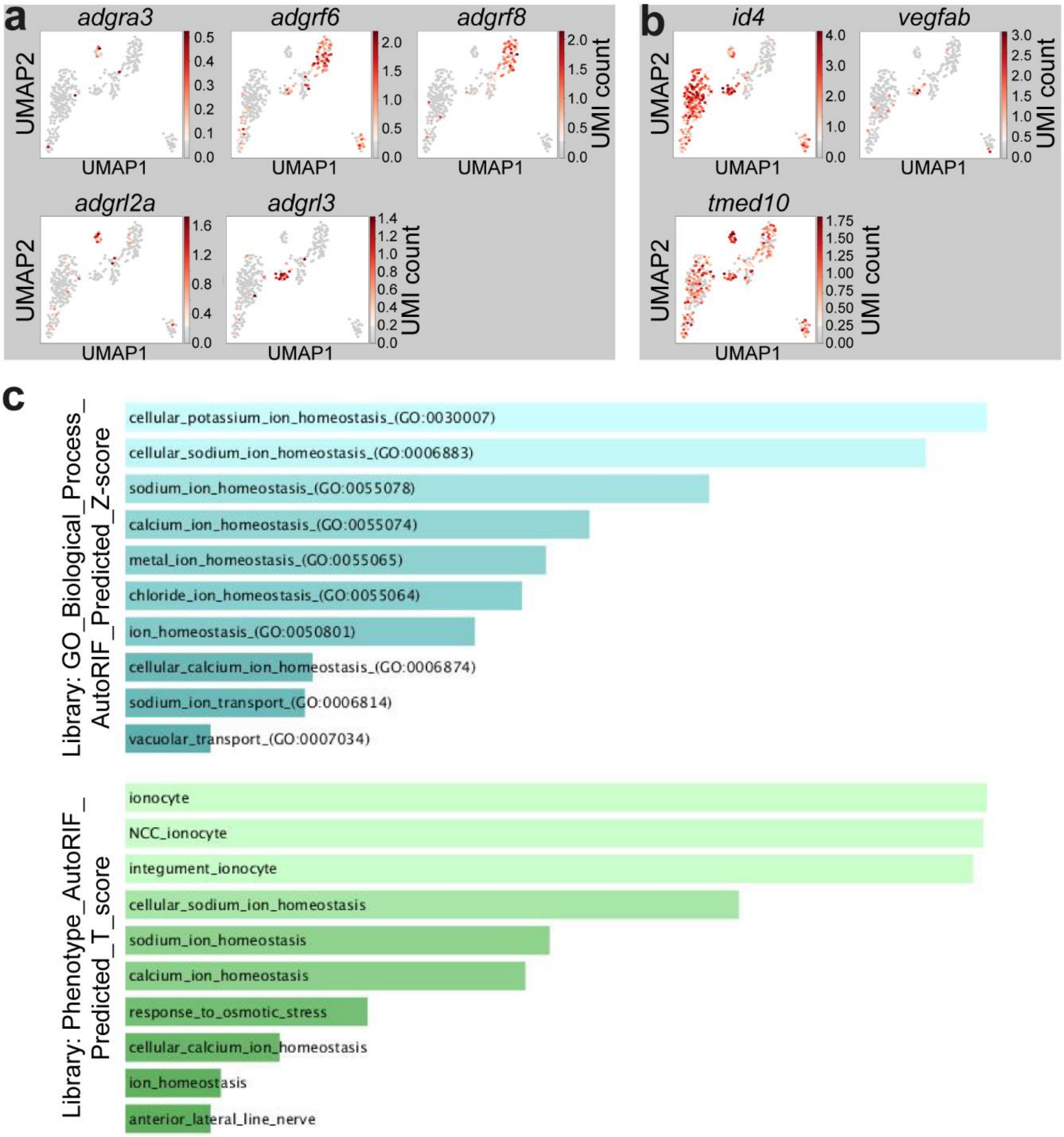

Figure S2. *gpr126* co-expression analysis. (**a, b**) UMAP plot of selected aGPCRs from Table S2 (a) and differentially expressed genes previously associated with Gpr126 selected from Table S3 (b), whereby the normalized and scaled UMI (Unique Molecular Identifier) counts are colored in a scale from 0 UMI counts (gray) to the highest UMI count (dark red). (**c**) FishEnrichr analysis of differentially expressed genes comparing cells in which *gpr126* was detected versus cells that lack *gpr126* UMIs (Table S4, top 10).
